# 

**Published:** 1860-03

**Authors:** 


### CONTENTS

OF THE

### Chicago Medical Journal for March, 1860.

ORIGINAL COMMUNICATIONS. PAGE.

Article 1.—Case of Extra Uterine Pregnancy, continuing three years and six months—fœtus removed by Gastrotomy. Reported by C. Goodbrake, M. D., Clinton, Ill... . 121

Article 2.—On the use of Opium. By Chas. Brackett, M. D. .......... 127

Article 3.—Case of a Tumor, apparently cancerous, disappearing suddenly. By T. B. Cox, M. D. .......... 130

Article 4.—Treatment of Indolent Ulcers—particularly those of a Lyphititic character. By B. Woodward, M. D. .......... 132

Article 5.—Periodicity of Intermittent Fever. By D. L. Robinson, M. D. .......... 134

Article 6.—Cases of Diphtheria. By J. J. Morgan, M. D .......... 137

SELECTIONS.

On the Diffiiculties and Advantages of Catheterism of the Air-Passages of the Chest. By Horace Green, M. D., LL. D., &c. .......... 140

Sulphurous Vapor Baths in Chronic Rheumatism .......... 160

Remarks on delivering the After-Birth .......... 162

Extract from the Speech of Gov. Wise, of Virginia .......... 165

Chronic Inversion of the Uterus .......... 166

Chloroform in Obstetrics .......... 168

EDITORIAL.

Book Notices. Practical Treatise on Fractures and Dislocations. By F. H. Hamilton, M. D. 170

Contributions to operative Surgery and Surgical Pathology. By J. M. Carnochau, M. D .......... 174

Therapeutics and Materia Medica. By Alfred Stille, M. D. .......... 175

Elements of Medical Jurisprudence. By T. R. and J. B. Beck .......... 177

Tranactions of the American Medical Association .......... 178

Introductory Lectures and Addresses on Medical Subjects. By G. B. Wood. 182

Pamphlet Notices .......... 169, 181

Rush Medical College .......... 183

National Society of Surgery .......... 183

